# Individual differences in threat and reward neural circuitry activation: Testing dimensional models of early adversity, anxiety and depression

**DOI:** 10.1111/ejn.15592

**Published:** 2022-01-19

**Authors:** Katherine S. Young, Camilla Ward, Meghan Vinograd, Kelly Chen, Susan Y. Bookheimer, Robin Nusslock, Richard E. Zinbarg, Michelle G. Craske

**Affiliations:** ^1^ Social, Genetic and Developmental Psychiatry (SGDP) Centre, Institute of Psychology, Psychiatry and Neuroscience King's College London London UK; ^2^ NIHR Maudsley Biomedical Research Centre King's College London London UK; ^3^ Center of Excellence for Stress and Mental Health Veterans Affairs San Diego Healthcare System San Diego California USA; ^4^ Department of Psychiatry University of California San Diego San Diego California USA; ^5^ Department of Psychology University of Arizona Tucson Arizona USA; ^6^ Department of Psychiatry and Biobehavioral Sciences University of California, Los Angeles (UCLA) Los Angeles California United States; ^7^ Department of Psychology Northwestern University Evanston Illinois USA; ^8^ The Family Institute Northwestern University Evanston Illinois USA; ^9^ Department of Psychology University of California, Los Angeles (UCLA) Los Angeles California USA

**Keywords:** anxiety, depression, early life adversity, face processing, fMRI

## Abstract

Altered functioning of the brain's threat and reward circuitry has been linked to early life adversity and to symptoms of anxiety and depression. To date, however, these relationships have been studied largely in isolation and in categorical‐based approaches. It is unclear to what extent early life adversity and psychopathology have unique effects on brain functioning during threat and reward processing. We examined functional brain activity during a face processing task in threat (amygdala and ventromedial prefrontal cortex) and reward (ventral striatum and orbitofrontal cortex) regions of interest among a sample (*N* = 103) of young adults (aged 18–19 years) in relation to dimensional measures of early life adversity and symptoms of anxiety and depression. Results demonstrated a significant association between higher scores on the deprivation adversity dimension and greater activation of reward neural circuitry during viewing of happy faces, with the largest effect sizes observed in the orbitofrontal cortex. We found no significant associations between the threat adversity dimension, or symptom dimensions of anxiety and depression, and neural activation in threat or reward circuitries. These results lend partial support to theories of adversity‐related alterations in neural activation and highlight the importance of testing dimensional models of adversity and psychopathology in large sample sizes to further our understanding of the biological processes implicated.

## Graphical abstract

A dimensional approach was used to reconsider relationships between childhood adversity, adolescent threat and reward brain functioning, and symptoms of anxiety and depression. Significant associations were found between more severe childhood deprivation adversity and greater activation of reward neural circuitry, but no significant effects associated childhood threat adversity with symptom dimensions of anxiety and depression, or threat neurocircuitry. Results partially support prior theories and highlight the importance of testing dimensional models in large samples to robustly examine theoretically implicated biological processes.

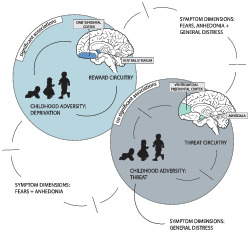

**Abbreviations**
BAS: Behavioral Activation Scale
BrainMAPD study: Brain Motivation and Personality Development study
CFA: confirmatory factor analysis
CTI: Childhood Trauma Interview
dACC: dorsal Anterior Cingulate Cortex
DSM‐5: Diagnostic and Statistical Manual of Mental Disorders—5th edition
EPQ‐N: Eysenck Personality Questionnaire—Neuroticism subscale
FEAT: FSL Expert Analysis Tool
fMRI: functional magnetic resonance imaging
FSL: FMRIB software library
OFC: orbitofrontal cortex
RDoC: research domain criteria
ROI: Region of interest
SCID‐5: Structured Clinical Interview for DSM‐5
vmPFC: ventromedial Prefrontal Cortex
VS: ventral striatum

## 1 INTRODUCTION

Childhood and adolescence are influential developmental periods for the brain (Casey et al., 2019; Schreuders et al., 2018) and psychopathology (Rohde et al., 2013). Early adversity is associated with the onset, maintenance, and exacerbation of common mental health problems, including depression (Rudolph et al., 2000; Wiersma et al., 2009) and anxiety (Heim & Nemeroff, 2001; Hovens et al., 2012). Rates of early adversity in the population are high, with approximately 20%–48% of individuals estimated to witness or experience emotional, physical or sexual abuse before the age of 16 years (Saunders & Adams, 2014), and up to 60% estimated to have experienced some other form of significant adversity (e.g., parental separation or emotional neglect) by the age of 18 years (Felitti et al., 1998; Hovens et al., 2012). Approximately 30% of all adult mental health disorders are estimated to have been preceded by experiences of early adversity (Kessler et al., 2010), which is also associated with earlier age of onset of mental health problems, poorer treatment response, increased risk of suicide, and more severe symptomatology (Hovens et al., 2012; Teicher & Samson, 2013).

A growing body of research implicates brain structure and function, particularly threat‐ and reward‐related brain circuitries, as an explanatory factor in the relationship between early adversity and mental health (McCrory et al., 2017). Yet, prior research is limited by being primarily focused on categorical investigations of early adversity and psychopathology, often independently of each other. Although early adversity has been shown to predict later mental health challenges, there is a vast degree of heterogeneity both in the type and severity of early adverse experiences and in mental health outcomes across individuals. Recent reviews have called for dimensionally based investigations of early adversity, fronto‐limbic brain functioning and mental health outcomes in order to better understand individual differences in risk and resilience to early adversity (Cohodes et al., 2020; VanTieghem & Tottenham, 2018). The current study aimed to examine the differential impact of dimensional measures of early adversity and anxiety and depression symptoms on the functioning of both threat and reward neural circuits in late adolescence.

### 1.1 Early adversity and threat system functioning

Early adversity has been linked to altered functioning of amygdala‐prefrontal circuitry, which is implicated in threat reactivity and the regulation of negative affect (McLaughlin et al., 2019; Morey et al., 2016). Across studies of children, adolescents and adults who experienced early adversity, there is consistent evidence of heightened amygdala reactivity to negative emotional facial expressions, in comparison with non‐affected peers (Dannlowski et al., 2012, 2013; Gee et al., 2013; Kraaijenvanger et al., 2020; McCrory et al., 2013; Tottenham et al., 2011; van Harmelen et al., 2013). Findings regarding the functional role of prefrontal regions (primarily medial or ventromedial prefrontal cortex) are more mixed. In groups of individuals who experienced early adversity, some studies have shown increased activation in regions of the prefrontal cortex when viewing emotional faces (Ganzel et al., 2013; Garrett et al., 2012; Godinez et al., 2016), whereas others have found decreased activation during encoding and retrieval of emotional words (van Harmelen et al., 2014), in comparison with individuals who had not experienced early adversity.

Altered reactivity of the brain's threat circuitry has been linked to anxiety disorders (Kujawa et al., 2015), suggesting a core neurobiological pathway through which early adversity may confer risk for later psychopathology. Similar to the effects observed among individuals exposed to early adversity, comparing groups of patients with and without anxiety disorders has demonstrated altered functioning of threat neurocircuitry in relation to anxiety. This includes heightened reactivity of the amygdala and dorsal anterior cingulate cortex (dACC) in response to threatening stimuli, coupled with altered functioning and connectivity patterns with prefrontal regulatory regions (Craske et al., 2017; Kim et al., 2011). A meta‐analysis of functional magnetic resonance imaging (fMRI) studies using face‐processing tasks to examine altered neural processing in anxiety disorders demonstrated consistent disruptions to activation in amygdala, as well as in prefrontal cortical regions thought to be important in the regulation of affective responses (Gentili et al., 2016).

### 1.2 Early adversity and reward system functioning

In addition to disruptions to threat circuitry, it has also been suggested that early adversity may increase the risk of future psychopathology through altered development of the brain's reward system, particularly the ventral striatum (VS) and orbitofrontal cortex (OFC) (Gerin et al., 2019; Kujawa et al., 2015; Novick et al., 2018; Pizzagalli, 2014). Exposure to early adversity has been associated with decreased activation of the VS when viewing emotional faces (Goff et al., 2013), consistent with a broader pattern of effects showing reductions in striatal activation during the anticipation or receipt of reward (Boecker‐Schlier et al., 2016; Mehta et al., 2009). Exposure to early life adversity has also been associated with reduced OFC activation during a reward‐based decision‐making task among children who had experienced maltreatment (Gerin et al., 2019).

Reduced, or ‘dampened’ functioning of the brain's reward system has been linked to major depressive disorder. Meta‐analyses of neuroimaging studies, which include studies of face processing, have identified altered activation in the VS and OFC during reward processing in individuals with depression (Keren et al., 2018; Ng et al., 2019). There is evidence of a particular association with anhedonia, a core symptom of depression, defined as the loss of interest or pleasure in previously enjoyed activities (American Psychiatric Association, 2013). Symptoms of anhedonia have been associated with reduced ventral striatal activation during the anticipation and receipt of reward (Forbes et al., 2009; Greenberg et al., 2015). Prior studies examining altered neural responding in depression using face processing tasks have typically involved small sample sizes, but the available evidence also demonstrates altered functioning of both VS (Fu et al., 2004; Surguladze et al., 2005) and OFC (Scheuerecker et al., 2010; Townsend et al., 2010).

### 1.3 Dimensional models of adversity and psychopathology

As reviewed elsewhere (Cohodes et al., 2020; VanTieghem & Tottenham, 2018), the majority of studies examining the impact of early adversity on altered brain function have focused on group‐based approaches. By categorising individuals into groups with and without adversity exposure, a potentially false dichotomy is created, with individuals who experienced mild or moderately severe adversity often falling into neither group, and important individual differences in responses to adversity being obscured. Although early adversity confers risk, many individuals are resilient and do not develop mental health problems following exposure to adversity. Dimensional approaches that capture individual differences in both the severity and type of adversity exposure offer the potential to better understand the nature of the relationship between adversity and brain function. One prominent dimensional model in this area differentiates between experiences of threat (in which physical wellbeing is at risk) and deprivation (in which there is an absence of required environmental inputs) (Sheridan & McLaughlin, 2014). Accumulating evidence indicates that early experiences of threat are linked to altered processing of negative stimuli in neural threat circuitry and symptoms of anxiety disorders (McLaughlin et al., 2019). Other studies have shown that experiences of deprivation are related to altered reward system functioning and symptoms of depression (Dennison et al., 2019; Hanson et al., 2015). Dimensional models of psychiatric symptoms have also been proposed to better understand continuous variance across broad domains of functioning in frameworks such as the Research Domain Criteria (RDoC; Insel et al., 2010). Dimensional models of anxiety and depression highlight symptom dimensions common to both anxiety and depression, as well as dimensions more specific to each diagnostic category (Clark & Watson, 1991; Prenoveau et al., 2010).

The current study uses a dimensional approach to investigate differential associations of early life adversity and symptoms of anxiety and depression with threat and reward neural circuitry functioning in late adolescence. We had two sets of hypotheses, based on prior literature and neurobiological theories of early life adversity, anxiety and depression. First, in line with prior research demonstrating altered threat circuitry activation in relation to early threat adversity, we hypothesised that early threat adversity severity and a symptom dimension related to anxiety would be associated with greater activation in the amygdala and reduced activation in the ventromedial prefrontal cortex (vmPFC) while viewing negative facial expressions (fearful and sad faces) compared with scrambled faces. Second, in line with theories regarding dampened reward circuitry activation in relation to early deprivation adversity, we hypothesised that deprivation adversity severity and a symptom dimension related to depression would be associated with lower activation in the VS and OFC when viewing positive facial expressions (happy faces) compared with scrambled faces.

## 2 METHODS

### 2.1 Participants

Participants were recruited for the Brain, Motivation and Personality Development (BrainMAPD) Study, a multi‐site longitudinal study that investigated positive and negative affective functioning in late adolescence to early adulthood. BrainMAPD was based at the University of California, Los Angeles (UCLA), and Northwestern University, however, only participants at the UCLA site completed the face processing task reported here. Participants were recruited through fliers and online advertising on university campuses and surrounding community areas. Due to the developmental focus of the BrainMAPD Study, inclusion criteria included being aged 18–19 years at the time of enrolment. Participants were initially screened on self‐reported trait neuroticism (Eysenck Personality Questionnaire‐Neuroticism, EPQ‐N; Eysenck & Eysenck, 1975) and reward sensitivity (Behavioral Activation Scale, BAS; Carver & White, 1994; for more details, see Young et al., 2020). Invited participants were selected based on their EPQ‐N and BAS scores, ensuring representation of low, mid and high tertiles on each scale. Exclusion criteria were as follows: magnetic resonance imaging (MRI) contraindications; not being right‐handed (assessed using the Edinburgh Handedness Inventory; Oldfield, 1971); not fluent in English; colour blindness; clinically significant substance use disorder in the last 6 months; lifetime symptoms of psychosis; lifetime symptoms of bipolar I disorder; use of antipsychotic medication (psychopathology‐based exclusion criteria were assessed using the Structural Clinical Interview for DSM‐5; First et al., 2015; SCID‐5).

A total of 115 participants completed the face processing task (at the UCLA study site only). Four individuals were excluded for technical difficulties during scanning; and 8 individuals were excluded for excessive motion (>10% outlier volumes, defined as 75th percentile +1.5 times interquartile range, based on framewise displacement, average of rotation and translation parameter differences, using weighted scaling (Power et al., 2012) as implemented in the FSLmotionoutliers function). One hundred three individuals are included in the current analyses (63% female; mean age at scan = 19.05 years, *SD* = .51). Participants were predominantly White (53%; 29% non‐Hispanic, 24% Hispanic) or Asian (39%), with a smaller proportion self‐reporting as Black (4%), multi‐racial (2%) or Native American (1%). Of the participants included here, 31 (30.10%) met criteria for a current anxiety disorder and 11 (10.68%) met criteria for a depressive disorder. A small number of individuals (3%) reported currently taking any medication for anxiety or depression (e.g., amitriptyline, lorazepam and sertraline). Ethical approval for this study was granted by the Institutional Review Board at UCLA and participants provided written, informed consent.

### 2.2 Measures

#### 2.2.1 Childhood Trauma Interview

The Childhood Trauma Interview (CTI; Fink et al., 1995) assesses six domains of adversity experienced prior to the age of 18 years: separation from or loss of a caregiver, physical neglect, emotional abuse or assault, physical abuse or assault, witnessing violence, and sexual abuse or assault. The CTI is a semi‐structured interview that retrospectively assesses childhood and adolescent adversity (birth to age 18 years), using standardised prompts to assess specific experiences within each domain of adversity, the age(s) during which it was experienced, and the frequency of occurrence. The CTI has been shown to be a reliable and valid instrument, exhibiting high inter‐rater reliability and convergent validity (Fink et al., 1995). Interviews were administered over the telephone by trained interviewers who subsequently rated each reported adversity on a scale of 1 (minimal or mild) to 6 (very extreme, sadistic). Summary scores were generated using a scoring system developed as part of our previous longitudinal study (the Youth Emotion Project; Vrshek‐Schallhorn et al., 2014) in which severity scores were calculated as the sum of the severity ratings for each reported adversity in each domain. We next created summary scores across two broad domains of adversity highlighted in a recent theoretical framework (Sheridan & McLaughlin, 2014). We created a ‘threat’ score, combining experiences characterised actual or threatened harm (summing scores for physical abuse or assault, witnessing violence, sexual abuse or assault, and emotional abuse domains; *α* = .54) and a ‘deprivation’ score, combining experiences characterised by the absence of expected care (summing the loss or separation from a caregiver and caregiver neglect domains; *α* = .47).

#### 2.2.2 Symptoms of anxiety and depression

Dimensional symptom measures of anxiety and depression were factor scores extracted from a dimensional model of anxiety and depression symptoms, the ‘trilevel model’ developed through exploratory and confirmatory factor (CFA) analyses in previous research (Kramer et al., 2019; Naragon‐Gainey et al., 2016; Prenoveau et al., 2010). The trilevel model is a hierarchical dimensional model that includes one broad factor common to both anxiety and depression (‘general distress’), as well as two intermediate factors: (i) ‘fears’, more common to anxiety disorders; and (ii) ‘anhedonia‐apprehension’ considered more common to depressive disorders (although symptoms of anhedonia are also common in some anxiety disorders, particularly generalised and social anxiety disorders; Brown et al., 1998; Kashdan, 2007).

Trilevel model factor scores were generated from 101 questionnaire items selected from self‐report symptom measures of anxiety and depression (for details, see Kramer et al., 2019; Young et al., 2020; and Table S1). As reported elsewhere (Kramer et al., 2019; Young et al., 2020), the CFA identified dimensions of general distress, fears and anhedonia‐apprehension, similar to findings in previous work. We extracted factor score estimates for these dimensions from the trilevel model for use in analyses presented here. Factor score estimates in the full BrainMAPD sample (*N* = 336; Kramer et al., 2019; Young et al., 2020) were quasi‐orthogonal and can consequently be considered statistically independent. Furthermore, within the sample reported here (*n* = 101) factor score estimates were not significantly correlated with each other (general distress and fears, *r*(101) = .11, *p* = .277; general distress and anhedonia‐apprehension, *r*(101) = −.07, *p* = .480; fears and anhedonia‐apprehension, *r*(101) = .13, *p* = .205).

### 2.3 Procedure and task

On the day of scanning, participants completed self‐report measures of anxiety and depression (described above) and then a 1‐h scanning session, including structural and functional MRI runs. The emotional expressions task consisted of passive viewing of female facial expressions (happy, sad, fearful, and neutral) and passive listening to emotional vocal expressions (note that emotional vocal expressions were presented separately to the facial expressions, and results from these stimuli are not reported here). Facial stimuli were obtained from the NimStim Set of Facial Expressions (Tottenham et al., 2009). Control stimuli were scrambled faces, created by applying fast Fourier transforms to amplitude and phase matrices of each image, resulting in an image with similar luminosity and frequency elements, but not recognisable as a face image (A. Hahn, personal communication, 2014). Although prior literature has predominantly used ‘neutral’ facial expressions as control stimuli, this practice is considered problematic as expressions intended to portray emotional neutrality can be interpreted differently by the viewer (Filkowski & Haas, 2017). This is a particular concern when examining processes related to anxiety and depression, conditions that are characterised by ‘negative interpretation bias’ of neutral or ambiguous stimuli (Yoon & Zinbarg, 2008). Stimuli were presented in blocks of 12 s, each containing six image presentations of the same expression type. Each image was 1 s in duration, and there was a jittered 0.5–1.5 s inter‐stimulus interval during which a fixation cross was presented. In total, 27 face stimulus blocks were presented, 3 of each stimulus type (happy, sad, fearful, neutral, and scrambled) as well as 12 ‘blank’ blocks (fixation cross only), which served as the implicit baseline in analyses. Stimulus order within block and overall block order was randomised across participants.

### 2.4 fMRI analysis

For fMRI acquisition parameters and preprocessing steps, see Supporting information. Analyses were conducted using the FMRIB software library (FSL) (Jenkinson et al., 2012). First‐level analyses included regressors of interest (face type: happy, sad, fearful, neutral and scrambled) and temporal derivatives, six motion regressors and additional regressors to censor outlying volumes (i.e., a regressor with a single time point corresponding to each outlier volume). Time‐series statistical analysis was carried out using FILM with local autocorrelation correction (Woolrich et al., 2001). Contrasts of interest were computed as follows: happy versus scrambled faces, fearful versus scrambled faces, sad versus scrambled faces and neutral versus scrambled faces. Second‐level whole brain analyses were conducted using single generalised linear models in FEAT (FMRI Expert Analysis Tool). Regressors were threat adversity severity, deprivation adversity severity, symptom dimension factor scores of general distress, fears and anhedonia‐apprehension, and sex (all mean‐centred). Resulting Z (Gaussianised T/F) statistic images were thresholded using a voxel‐wise cluster‐defining threshold of *z* = 3.1 (*p* < .001) and cluster correction threshold of *p* < .05. Region of interest (ROI) analyses were conducted on a priori ROIs for bilateral amygdala, VS, vmPFC and OFC (note that the vmPFC ROI was non‐overlapping with OFC ROIs, see Supporting information for details).

### 2.5 Statistical analyses

First, relationships between dimensional measures of adversity and symptom factor scores were examined using Spearman's rho correlations. Whole brain analyses (described above) examined relationships between neural activation to face stimuli and measures of adversity and anxiety and depression symptoms. Next, to examine threat and reward circuitry engagement among a priori ROIs, *t* tests comparing each face type versus scrambled face viewing were conducted across all ROIs. Intraclass correlations were computed on ROI parameter estimates for three emotional expression face types (fearful, sad and happy), demonstrating reliability of .48–.56 (with the exception of right VS, which had a substantially lower estimate of .33; see Supporting information, Table S2 for details). Multilevel analyses were conducted on ROI data to examine the unique effects of dimensional measures of threat adversity, deprivation adversity, general distress, fears and anhedonia‐apprehension on threat and reward circuitry functioning (lme4 package [Bates et al., 2014] in R [R Core Team, 2020]). Multilevel models (one for threat ROIs [amygdala and vmPFC] and one for reward ROIs [VS and OFC]) in a two‐level hierarchical data structure were computed independently for each contrast. Individual ROIs were entered at Level 1 and participant at level 2. Dimensional measures of adversity (threat and deprivation severity), anxiety and depression symptoms (general distress, fears and anhedonia‐apprehension) as well as participant sex were included as predictors.

The R oslrr package (Hebbali, 2020) was used to examine assumptions of statistical models used, including Kolmogorov–Smirnoff tests to examine the normality of the distribution of all variables and the distribution of model residuals using Q–Q plots. Results of these analyses (see Supporting information, Table S3) indicated presence of outliers in threat and deprivation adversity variables, so analyses were repeated with and without outliers.

## 3 RESULTS

### 3.1 Dimensional measures of early adversity, anxiety and depression

Correlation analyses demonstrated a significant association between threat adversity severity (*M* = 8.53, *SD* = 7.54, range of scores observed = 0–39) and deprivation adversity severity (*M* = 4.71, *SD* = 4.74, range of scores observed = 0–28) with a medium effect size (Table 1). Correlation analyses between adversity severity scores and dimensional symptom factor scores for anxiety and depression demonstrated a small significant association between threat adversity severity and general distress (Table 1). There were no significant associations with deprivation adversity severity, or with fears or anhedonia‐apprehension scores.

**TABLE 1:** Correlation coefficients (Spearman's rho) and significance levels for associations between dimensional measures of adversity and anxiety/depression

	Deprivation severity		Threat severity	
	*r*_*s*_	*p*	*r*_*s*_	*p*
Threat severity	.47	<.001		
General distress	.08	.400	.24	.015
Fears	.03	.732	.05	.596
Anhedonia‐apprehension	−.07	.490	‐.10	.339

*Note*: Spearman's correlations were conducted due to the positively skewed distribution of adversity measures.

### 3.2 Faces task activation

#### 3.2.1 Whole brain analyses

Overall, passive viewing of facial expressions versus scrambled faces was associated with activation in visual cortex and temporal lobe brain regions (see Table S4). Viewing of happy facial expressions was additionally associated with small clusters of activation among prefrontal brain regions.

#### 3.2.2 ROI analyses

**FIGURE 1:**
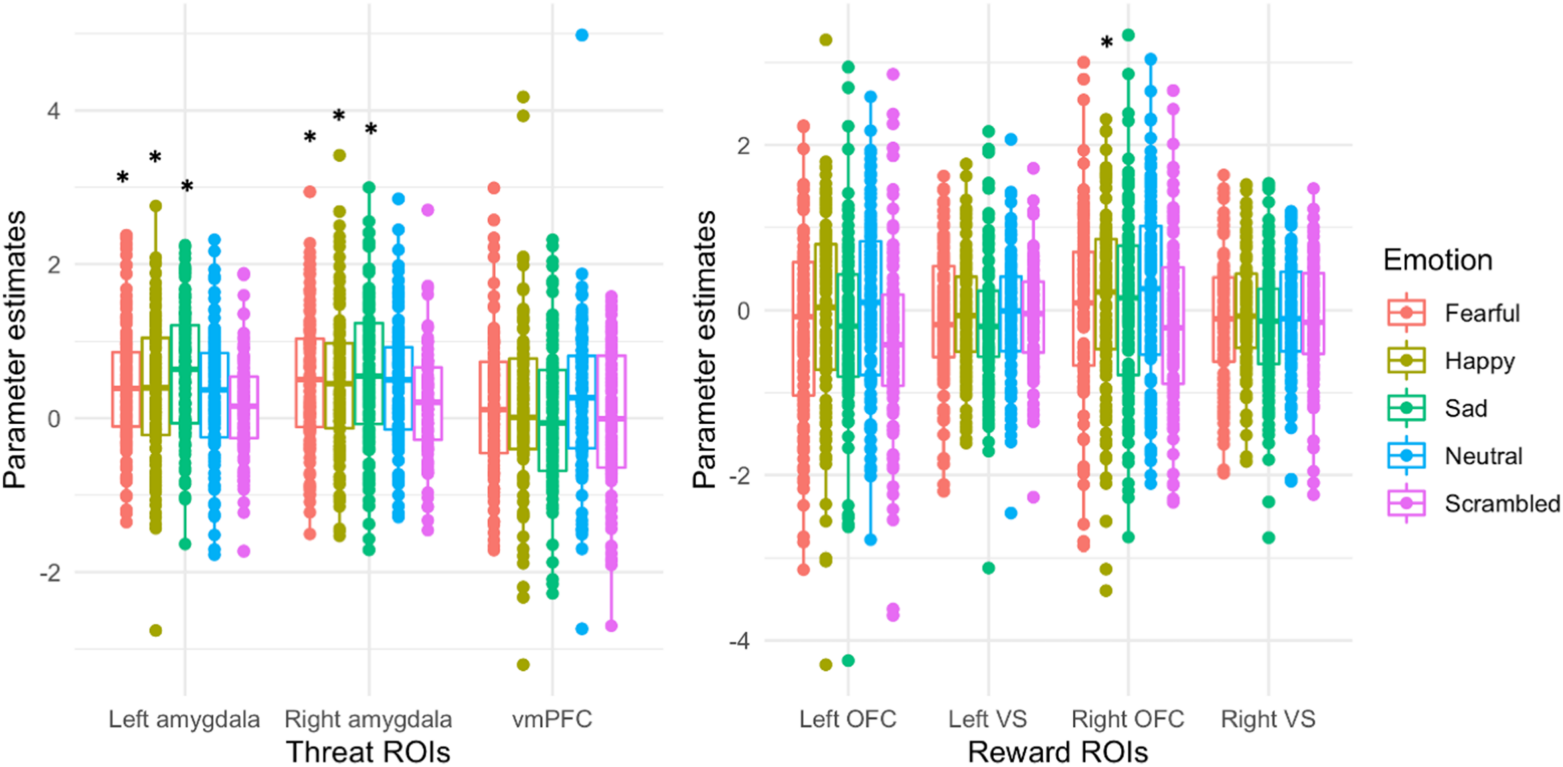
Activation across threat (upper) and reward (lower) regions of interest (ROIs) in response to emotional faces (fear, sad and happy) compared with neutral and scrambled faces. * denotes a significant difference relative to scrambled faces (*p* < .05)

Contrasts of facial expressions versus scrambled face images demonstrated significant activation of threat circuitry ROIs and more limited activation of reward circuitry ROIs. Specifically, among threat circuitry ROIs, there was greater activation in bilateral amygdala for fearful, happy and sad faces (vs. scrambled faces). Among reward circuitry ROIs, there was less deactivation in left OFC when viewing happy versus scrambled faces (Figure 1).

### 3.3 Adversity, symptoms and neural activation

A series of multilevel analyses were conducted to examine the unique variance associated with adversity and symptom dimensions and neural circuitry activation. Models were computed for each face contrast, adding general distress, anhedonia‐apprehension and fears variables simultaneously, as well as either threat or deprivation adversity scores. As the threat and deprivation variables were significantly correlated with a medium effect size, adversity scores were entered one at a time was entered into each mode. Symptom dimension variables were entered simultaneously as they are quasi‐orthogonal.

### 3.4 Threat adversity, anxiety symptoms and threat circuitry activation

We examined threat adversity and anxiety symptoms as moderators of threat circuitry activation during viewing of fearful versus scrambled and sad versus scrambled faces. We initially demonstrated a significant association between threat adversity and threat circuitry activation when viewing fearful versus scrambled faces (estimate = .02 [.00, .04], *p* = .022). However, after excluding two data points with outlier values on the upper end of the threat adversity measure, this effect was no longer statistically significant (estimate = .02 [−.00, .04], *p* = .139). There were no significant effects of threat adversity scores on threat circuitry activation during viewing of sad versus scrambled faces. There were no significant effects of symptom dimensions on activation of threat circuitry during viewing of either fearful or sad (versus scrambled) faces (see Table 2).

### 3.5 Deprivation adversity, depression symptoms and reward circuitry activation

**FIGURE 2:**
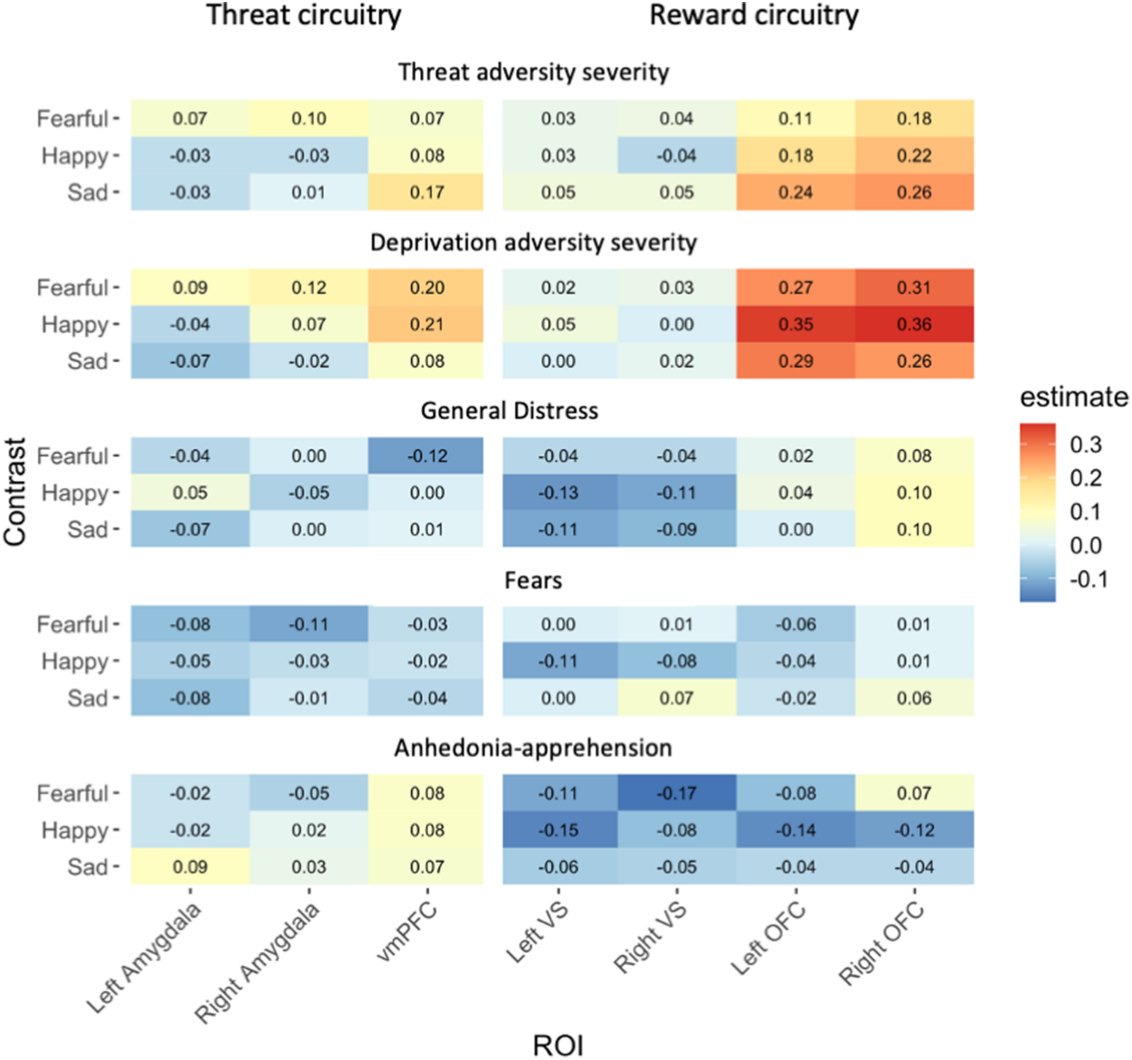
Spearman's correlations (rho) of dimensional factors (adversity: threat and deprivation; symptoms: general distress, fears and anhedonia‐apprehension) with activation of threat and reward circuitry for each face (vs. scrambled) contrast

We examined deprivation adversity and depression symptoms as moderators of reward circuitry activation during viewing of happy versus scrambled faces. We observed a significant effect of deprivation adversity scores on activation of reward circuitry (estimate = .03 [.00–.06], *p* = .026), which remained statistically significant after exclusion of 13 participants with outlier scores on the deprivation adversity measure (estimate = .04 [.00, .08], *p* = .036). Examining effect sizes with individual ROIs there were small‐to‐medium effect sizes for left and right OFC (*r* = .35–.36) and minimal to no effects in VS (*r* = .00–.05; Figure 2) for the happy versus scrambled comparison. There were no significant effects of symptom dimensions of general distress, fears or anhedonia‐apprehension on reward circuitry activation (see Table 2).

### 3.6 Exploratory analyses

We hypothesised that differences in threat and reward circuitry activation would be specific to certain facial types (fearful and sad for threat circuitry; happy for reward circuitry). However, ROI analyses demonstrated a lack of differentiation of the facial expression types that activated threat and reward circuitry. Therefore, we conducted additional exploratory analyses to examine whether there were differences in neural responses to face stimuli not directly specified in the hypotheses, namely, happy faces in relation to functioning of threat circuitry, and sad or fearful faces in relation to functioning of reward circuitry. None of these analyses resulted in any statistically significant effects (see Table 2).

**TABLE 2:** Multilevel model estimates for each face type, separated by threat and reward circuitry regions of interest (ROIs) and by adversity dimension (threat or deprivation)

	Fear > scrambled		Sad > scrambled		Happy > scrambled	
	Estimate	*p*	Estimate	*p*	Estimate	*p*
Threat circuitry ROIs						
(Intercept)	.18	.183	.37	.006*	.22	.132
Adversity: threat	.02	.139	.01	.379	.00	.804
General distress	−.08	.252	−.04	.584	−.00	.959
Anhedonia‐apprehension	.03	.732	.02	.784	.02	.784
Fears	−.13	.083	−.08	.240	−.06	.457
Gender	−.15	.272	−.16	.236	−.13	.354
Reward circuitry ROIs						
(Intercept)	.11	.432	.00	.981	.19	.511
Adversity: deprivation	.03	.078	.03	.074	.04	.036*
General distress	−.01	.908	−.07	.361	−.04	.603
Anhedonia‐apprehension	−.05	.500	−.13	.141	−.16	.082
Fears	.02	.797	.03	.679	.00	.971
Gender	−.24	.077	−.17	.253	−.16	.310

*Note*: Effects presented here are with outliers removed (see Supporting information, Table S4 for results prior to omitting outliers).

* *p* < .05.

## 4 DISCUSSION

Using dimensional measures of early life adversity and symptoms of anxiety and depression, we examined individual differences in activation of threat (amygdala and vmPFC) and reward (VS and OFC) brain regions. In relation to threat neurocircuitry, we hypothesised that threat adversity severity and symptom dimensions related to anxiety would be associated with greater amygdala and reduced vmPFC activation during viewing of fearful and sad faces. We observed no statistically significant effects to support this hypothesis that were robust to the removal of outlier values on the threat adversity variable. In relation to reward neurocircuitry, we hypothesised that deprivation adversity severity and symptom dimensions related to depression would be associated with reduced activation in the VS and OFC. Partially supporting this hypothesis, we observed an association between deprivation adversity and reward circuitry activation during viewing of happy versus scrambled faces. We observed no significant associations between depression symptom dimensions and reward circuitry activation.

The multilevel modelling approach used indicated a significant association between deprivation adversity and reward‐circuitry (VS, OFC) activation, and post‐hoc examination of effect sizes indicated that the strongest effects observed were in left and right OFC. However, these effects were in the opposite direction to that hypothesised, with higher levels of deprivation adversity associated with *greater* activation in OFC. We hypothesised blunted OFC activation due to previous findings demonstrating reduced OFC activation among adolescents who experienced maltreatment in a reinforcement learning task (Gerin et al., 2017), consistent with findings from depression literature that associate blunted reward sensitivity with elevated depression symptoms (Scheuerecker et al., 2010; Townsend et al., 2010). However, developmental literature points to a heightened sensitivity to rewarding cues in adolescence (Galván, 2010) and studies of social anxiety disorder suggest a developmental transition from hyper‐responsiveness to social cues in adolescence, shifting to blunted social responding in adulthood (Guyer et al., 2012; Richey et al., 2019). In addition, a recent meta‐analysis of reward circuitry in depression demonstrated hyper‐reactivity in the OFC, thought to be indicative of maladaptive regulation of striatal regions (Ng et al., 2019). Heightened OFC activation in response to happy faces may therefore be indicative of elevated adolescent sensitivity to rewarding stimuli among individuals who experienced greater childhood deprivation, which may also transition with development into blunted responses in adulthood. Alternatively, heightened OFC activation may be indicative of maladaptive regulation among reward circuitry. Longitudinal studies tracking the same individuals across this developmental transition using regulation‐focused tasks would allow direct investigation of these alternative explanations. Additionally, although the OFC is frequently discussed in the context of reward, it is known to be involved in representation of the subjective value of stimuli, both positively and negatively valenced (Berridge & Kringelbach, 2013; Rich & Wallis, 2016), so altered sensitivity may reflect a more general adjustment in the subjective valuation of stimuli.

A lack of other significant associations between dimensional measures of adversity, psychopathology and brain function was surprising in the context of prior findings demonstrating group‐based differences in neural responses during face‐processing tasks. However, recent literature examining dimensional models of psychopathology have also failed to identify robust markers of emotional or psychological processing that are consistent across units of analysis (Eisenberg et al., 2019; Peng et al., 2021). The ROIs examined here represented a small group of brain regions, which may have prevented detection of effects in other regions (although whole brain analyses did not demonstrate robust effects). The rationale behind the selection of the current set of ROIs was to specifically examine regions most often discussed as being implicated in the processing of ‘threat’ and ‘reward’ cues, and previously shown to have relationships with group‐differences in adversity and anxiety/depression symptomatology. We sought to limit the number of ROIs included in order to minimise type I error. Arguably, inclusion of other ROIs, such as the anterior cingulate cortex, considered a central hub integrating emotional, cognitive and social information (Lichenstein et al., 2016), may have allowed detection of additional effects. Below we discuss a number of additional potential factors that may influence results shown here, in comparison with prior work.

In our sample, we observed limited relationships between adversity and symptom dimensions. The dimension of general distress was significantly associated with the threat adversity dimension with a small effect size, but there were no significant associations between fears or anhedonia‐apprehension and either threat or deprivation adversity. One possible explanation for this may be related to the stress‐acceleration hypothesis. This hypothesis states that changes in neural functioning following exposure to early adversity may be adaptive in the short‐term but may contribute towards heightened vulnerability for psychopathology in the long‐term (Callaghan & Tottenham, 2016). Participants in this study were aged 18–19 years at the time of scanning, so it may be that although some changes in neural functioning related to early adversity were observed, these had not yet fully contributed to anxious or depressive symptomatology.

Alternatively, whereas early adversity is a known risk factor for later psychopathology, many individuals who experience adversity are resilient and do not subsequently develop anxiety or depression. As participants were recruited as part of a larger longitudinal study, the broader goal of which did not focus on adversity, and were mostly college students at large universities, it may be that the current sample was biased towards higher levels of resilience. Although there was some evidence of associations between early adversity and altered neural reactivity to emotional cues, it may be that compensatory processes that contribute to resilience prevent these disruptions from manifesting in symptoms of depression or anxiety. It is also possible that effects were smaller in magnitude than we were powered to detect with the current sample. Further exploration of these possibilities would be of interest in relation to longer‐term follow‐up of this sample.

Passive face viewing tasks have been used in prior literature examining group differences in brain functioning across individuals with and without adversity or anxiety/depression. However, a passive task may not be optimal for the examination of disrupted cognitive or affective processes associated with depression and anxiety (Infantolino et al., 2018). A task requiring explicit appraisal or interpretation of facial expressions may be more effective at engaging circuits implicated in negative biases considered to be a core cognitive feature of anxiety and depression (Mathews & MacLeod, 2005). In addition to disruptions in emotion *responsivity*, disruptions to emotion *regulation* (the capacity to manage or control emotions) are a key feature of anxiety and depressive disorders (Young et al., 2020). A passive viewing task is not optimal for separating potential disruptions to emotion regulation from emotion responsivity, potentially masking important differences in these separable neural processes.

Some additional limiting factors may also have reduced the ability to detect robust associations. First, due to the longitudinal nature of the larger study, the age range of participants sampled was limited to 18–19 years. Although this provides a focused age group in which to examine the effects of interest, this may limit comparisons with other age groups, particularly as both threat and reward circuitries undergo further development throughout late adolescence and early adulthood (Casey et al., 2019). In addition, as mentioned above, participants in this study were not selected on the basis of their scores on early adversity measures. While the overall mean adversity experienced by participants in the current sample was relatively low, a broad range of scores was observed in the present study, comparable with that found in prior work demonstrating associations between childhood adverse experiences and altered functional brain activity (Dannlowski et al., 2012, 2013). We aimed to examine separable dimensions of early adversity based on prominent theories of the neurobiological impact of threat and deprivation (Sheridan & McLaughlin, 2014). However, internal consistency of threat and deprivation adversity in the current sample was modest, and alternative quantifications of dimensional aspects of adversity (e.g., child's age at the time of exposure to adversity [Zhu et al., 2019], controllability of stressor [Cohodes et al., 2020; VanTieghem & Tottenham, 2018]) might have better psychometric properties and contribute to further understanding of these relationships. Poor measurement reliability is also a particular concern in task‐based fMRI activation estimates (Infantolino et al., 2018). Reliability assessed here was in a reasonable range (~.50), however, future optimisation of functional tasks to maximise reliability will be essential to examine individual difference effects on functional brain activity with greater confidence (Elliott et al., 2020). Finally, although findings presented here were statistically significant, effect sizes were relatively small, indicating that early life adversity accounts for only a small amount of overall variance observed in reward circuitry functioning during socio‐emotional processing.

In conclusion, we observed limited associations between dimensional measures of adversity, symptoms of anxiety and depression, and functioning of neural threat and reward circuits. Using multilevel modelling, we observed an association between the severity of deprivation adversity and activation of reward circuitry during passive viewing of happy faces. This aligns with one prominent neurobiological theory of stress, which suggest that early life adversity may impact reward system functioning in the brain (Pizzagalli, 2014). However, we failed to identify significant relationships with threat circuitry activation, despite prior work indicating between‐group differences in functioning of this circuit in relation to early life adversity and anxiety and depressive disorders. Overall, our findings demonstrate only partial support of a dimensional model of early life adversity on neurobiological processing of emotional cues. Replication of this type of dimensional approach will be important to further examine the robustness of previously identified neural processes in a potential causal pathway from early adversity to the development of psychopathology. Future work using fMRI tasks that elicit more specific cognitive processes known to be implicated in the development and maintenance of anxiety and depressive disorders may allow a more targeted investigation of altered emotional functioning at a neurobiological level.

## CONFLICT OF INTEREST

The authors declare no conflicts of interest.

## AUTHOR CONTRIBUTIONS

MGC, RN, REZ and SYB conceptualised the overall project from which the data were drawn. KSY conceptualised the current research question and analysis. KSY, MV and KC collected the data; KSY and CW conducted data analyses and wrote the manuscript. All authors edited the manuscript.

### PEER REVIEW

The peer review history for this article is available at https://publons.com/publon/10.1111/ejn.15592.

## Supporting information


**Table S1**. Details of items from symptom measures contributing to trilevel dimensional model
**Figure S1**. ROI images displayed on a template (MNI) brain. Yellow = vmPFC; red = right OFC; dark blue = left OFC; green = ventral striatum; pale blue = amygdala.
***Table S2***. Internal consistency (kappa) from intraclass correlations examining the reliability of ROI activation across emotional face types. Estimates are provided both for contrasts of emotional vs. scrambled faces (fear vs. scrambled; sad vs. scrambled; happy vs. scrambled) and for emotional faces alone (fear, sad or happy versus implicit baseline)
***Table S3***. Results of Kolomogorov‐Smirnoff tests of normality for variables used in multilevel modelling analyses
***Table S4***. Whole brain analyses for the main effect of each face versus scramble face contrast and the association with dimensional measures of adversity and anxiety/depression symptoms
***Table S4***. Multi‐level model estimates for each face type, separated by threat and reward circuitry ROIs and by adversity dimension (threat or deprivation), * *p* < 0.05. Effects presented here are *including* outliers

## Data Availability

ROI data and analysis code is available online (https://osf.io/wjm5d/).
