# Supplementary material for: Individual differences in threat and reward neural circuitry activation: Testing dimensional models of early adversity, anxiety and depression
Source: Eur J Neurosci. 2022 Jan 19;55(9-10):2739–53. doi: 10.1111/ejn.15592 (PMC9149108; doi:10.1111/ejn.15592)
Supplement: Supplementary file 1 — Table S1. Details of items from symptom measures contributing to trilevel dimensional model Figure S1. ROI images displayed on a template (MNI) brain. Yellow = vmPFC; red = right OFC; dark blue = left OFC; green = ventral striatum; pale blue = amygdala. Table S2 . Internal consistency (kappa) from intraclass correlations examining the reliability of ROI activation across emotional face types. Estimates are provided both for contrasts of emotional vs. scrambled faces (fear vs. scrambled; sad vs. scrambled; happy vs. scrambled) and for emotional faces alone (fear, sad or happy versus implicit baseline) Table S3 . Results of Kolomogorov‐Smirnoff tests of normality for variables used in multilevel modelling analyses Table S4 . Whole brain analyses for the main effect of each face versus scramble face contrast and the association with dimensional measures of adversity and anxiety/depression symptoms Table S4 . Multi‐level model estimates for each face type, separated by threat and reward circuitry ROIs and by adversity dimension (threat or deprivation), * p < 0.05. Effects presented here are including outliers [file EJN-55-2739-s001.docx]

Supplementary Materials

***Table S1.*** Details of items from symptom measures contributing to trilevel dimensional model

| Questionnaire name | Acronym | Total items | Items used | Original reference |
| --- | --- | --- | --- | --- |
| Fear Survey Schedule-II | FSS | 50 | 7 | Geer, 1965 |
| Albany Panic and Phobia Questionnaire | APPQ | 22 | 10 | Rapee et al., 1994 |
| Social Phobia Scale (self-consciousness subscale) | SPS | 13 | 8 | Mattick & Clarke, 1998; Zinbarg & Barlow, 1996 |
| Inventory to Diagnose Depression | IDD | 21 | 8 | Zimmerman et al., 1986 |
| Mood and Anxiety Symptom Questionnaire | MASQ | 90 | 34 | Watson et al., 1995 |
| Penn State Worry Questionnaire | PSWQ | 16 | 16 | Meyer et al., 1990 |
| Obsessive-Compulsive Inventory Revised | OCI-R | 18 | 18 | Foa et al., 2002 |

*fMRI Data Acquisition*

Imaging data was acquired on Prisma 3.0 Tesla whole-body scanner using a 64-channel head coil (Siemens Medical Systems, Iselin, New Jersey) at the UCLA Ahmanson-Lovelace Brain Mapping Centre. High resolution structural images (T1-weighted) were acquired using a magnetized prepared rapid acquisition gradient echo (MPRAGE) sequence using 0.8mm isotropic voxels, TR/TE/flip angle=2300ms/2.99ms/7°, FOV= 256mm², 208 slices. Blood oxygenation level-dependent (BOLD) functional images were acquired using 2mm isotropic voxels, TR/TE/flip angle=720ms/37ms/52°, FOV = 208 mm², 72 slices, 795 volumes, multiband acceleration factor 8, auto-aligned. Total acquisition time (TA) was 9 minutes 32 seconds.

*fMRI Preprocessing*

Raw dicom files were converted to NIFTI format using dcm2nii (MRIcroN, <http://www.cabiatl.com/mricro/mricron/dcm2nii.html>). Data were processed and analysed using FSL (FMRIB’s Software Library, [www.fmrib.ox.ac.uk/fsl](http://www.fmrib.ox.ac.uk/fsl)). Structural data was corrected for spatial intensity variations (bias field correction) using FAST (FMRIB's Automated Segmentation Tool; Zhang et al., 2001), and brain extraction was performed using optiBET (optimized brain extraction; Lutkenhoff et al., 2014).

Functional data were brain extracted using BET (Brain Exraction Tootl, FSL; Smith, 2002) and bias field corrected using N4BiasFieldCorrection, run twice (ANTS registration suite; Tustison et al., 2010). Remaining processing was carried out using FEAT (FMRI Expert Analysis Tool) Version 6.00. Registration to high resolution structural space images was carried out using FLIRT (Jenkinson & Smith, 2001; Jenkinson, 2002). Registration from high resolution structural to standard space was then further refined using FNIRT (nonlinear registration; Andersson et al., 2007). The following pre-statistics processing was applied: motion correction using MCFLIRT (Jenkinson, 2002), slice-timing correction using Fourier-space time-series phase-shifting, spatial smoothing using a Gaussian kernel of FWHM 4.0mm, grand-mean intensity normalisation of the entire 4D dataset by a single multiplicative factor, and high-pass temporal filtering (0.01Hz) to remove low frequency artifacts.

Amygdala and ventral striatum ROIs were defined anatomically: for the amygdala, Harvard-Oxford probabilistic atlas amygdala masks were thresholded at >50% probability and binarized; for the ventral striatum, images from the Oxford-GSK-Imanova structural atlas were used (Tziortzi *et al.*, 2011). vmPFC and OFC ROIs were functionally-defined based on recent meta-analyses of threat and reward processing. The vmPFC ROI was based on a sphere (5mm radius) around peak vmPFC activation (MNI coordinates: x = -2; y = 56; z = -14) reported in a meta-analysis of human fear conditioning (Fullana *et al.*, 2016). The OFC ROIs were based on spheres (5mm radius) around peak left and right OFC activation (MNI coordinates left: x = -2; y = 42; z = -6; right: x = 2, y = 44, z = -10) reported in a meta-analysis of monetary reward (Oldham *et al.*, 2018).


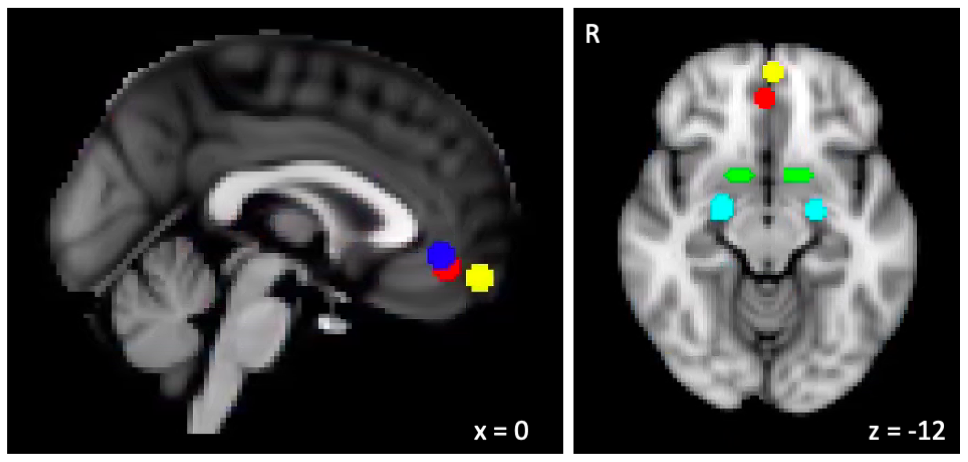


Figure S1. ROI images displayed on a template (MNI) brain. Yellow = vmPFC; red = right OFC; dark blue = left OFC; green = ventral striatum; pale blue = amygdala.

*Internal consistency of fMRI activation*

Internal consistency of fMRI activation was estimated by calculating the intraclass correlations of activation estimates for each ROI, across stimulus type (emotional faces: fear, sad and happy; Table S2). Prior examination of internal consistency has suggested that reliability is higher for individual stimulus estimates, relative to contrast scores. We computed intraclass correlations both for contrast (face vs. scrambled face) and individual stimulus (face vs. implicit baseline) values. Kappa values were higher in our data for contrast (face vs. scrambled face) images, so these were used in analyses presented.

***Table S2.*** Internal consistency (kappa) from intraclass correlations examining the reliability of ROI activation across emotional face types. Estimates are provided both for contrasts of emotional vs. scrambled faces (fear vs. scrambled; sad vs. scrambled; happy vs. scrambled) and for emotional faces alone (fear, sad or happy versus implicit baseline)

| ROI | Contrast score: emotional face vs. scrambled face | Single condition score: emotional face vs. implicit baseline |
| --- | --- | --- |
| vmPFC | 0.48 | 0.31 |
| Left amygdala | 0.52 | 0.40 |
| Right amygdala | 0.56 | 0.49 |
| Left ventral striatum | 0.50 | 0.29 |
| Right ventral striatum | 0.33 | 0.30 |
| Left OFC | 0.48 | 0.30 |
| Right OFC | 0.49 | 0.27 |

*Normality testing of predictors and residuals*

Assumption testing for models used was examined using: 1) Kolmogorov-Smirnoff (K-S) tests of normality, 2) scatterplots of residuals vs. fitted values to assess linearity, and 3) distribution of residuals using Q-Q plots. K-S tests demonstrated that symptom dimension variables and fMRI contrast scores were all normally distributed (see Table S3). Both adversity severity dimensional scores were significantly non-normally distributed, with a positive skew. Analyses were therefore repeated with and without outlier values for these variables.

***Table S3.*** Results of Kolomogorov-Smirnoff tests of normality for variables used in multilevel modelling analyses

| **Variable group** | **Variable** | **D (*p*)** |
| --- | --- | --- |
| Dimensional adversity variables | Threat | .78 (< .001) |
|  | Deprivation | .70 (< .001) |
| Dimensional symptom scores | General Distress | .08 (.400) |
|  | Fears | .06 (.886) |
|  | Anhedonia-apprehension | .08 (.497) |
| fMRI contrast variables | Threat circuitry: Fear vs. scrambled | .34 (.867) |
|  | Threat circuitry: Sad vs. scrambled | .03 (.991) |
|  | Threat circuitry: Happy vs. scrambled | .05 (.320) |
|  | Reward circuitry: Fear vs. scrambled | .02 (.990) |
|  | Reward circuitry: Sad vs. scrambled | .05 (.204) |
|  | Reward circuitry: Happy vs. scrambled | .03 (.893) |

***Table S4.*** Whole brain analyses for the main effect of each face versus scramble face contrast and the association with dimensional measures of adversity and anxiety/depression symptoms

| Main Effect | Anatomical region | Coordinates (MNI) | | | Cluster  size (k) | Z-Max | P-value |
| --- | --- | --- | --- | --- | --- | --- | --- |
|  |  | x | y | z |  |  |  |
| **Fear > scrambled** | |  |  |  |  |  |  |
| *Main effect* | Anterior cingulate cortex | 4 | -34 | 22 | 1952 | 5.35 | < .001 |
|  | Angular gyrus | 42 | -54 | 24 | 658 | 4.42 | < .001 |
|  | Lateral occipital cortex | -52 | -66 | 14 | 140 | 5.09 | .002 |
|  | Supramarginal gyrus | -54 | -40 | 36 | 108 | 4.04 | .008 |
|  | Parietal operculum cortex / Supramarginal gyrus | 56 | -24 | 24 | 91 | 3.95 | .019 |
|  | Anterior cingulate cortex | 4 | 20 | 30 | 76 | 4.01 | .045 |
| *Association with dimensional measures* | No suprathreshold clusters |  |  |  |  |  |  |
|  |  |  |  |  |  |  |  |
| **Happy > scrambled** |  |  |  |  |  |  |  |
| *Main effect* | Precuneous cortex | 4 | -66 | 24 | 3768 | 6.02 | < .001 |
|  | Lateral occipital cortex, superior division | -38 | -68 | 32 | 474 | 4.67 | < .001 |
|  | Frontal Pole | -34 | 38 | 14 | 403 | 5.16 | < .001 |
|  | Lateral occipital cortex, superior division | 48 | -64 | 38 | 367 | 4.64 | < .001 |
|  | Anterior cingulate cortex | 0 | 32 | 16 | 137 | 4.41 | .002 |
|  | Right hippocampus | 30 | -26 | -12 | 98 | 4.43 | .016 |
|  | Parietal operculum cortex / Supramarginal gyrus | -50 | -42 | 28 | 97 | 4.02 | .017 |
|  | Middle temporal gyrus | 64 | -8 | -24 | 87 | 3.97 | .028 |
|  | Middle frontal gyrus | 34 | 24 | 46 | 86 | 3.89 | .030 |
|  | Left hippocampus / left thalamus | -20 | -38 | -4 | 79 | 4.47 | .044 |
| *Association with dimensional measures* | No suprathreshold clusters |  |  |  |  |  |  |
|  | |  |  |  |  |  |  |
| **Sad > scrambled** | |  |  |  |  |  |  |
| *Main effect* | Precuneous cortex | 2 | -64 | 26 | 1221 | 4.93 | < .001 |
|  | Right amygdala / Right hippocampus | -18 | -4 | -20 | 257 | 4.69 | < .001 |
|  | Temporal fusiform cortex | -42 | -36 | -26 | 94 | 4.65 | .015 |
|  | Temporal occipital fusiform cortex | 44 | -44 | -24 | 84 | 4.69 | .025 |
| *Association with dimensional measures* | No suprathreshold clusters |  |  |  |  |  |  |
|  |  |  |  |  |  |  |  |
| **Neutral > scrambled** |  |  |  |  |  |  |  |
| *Main effect* | Precuneous cortex | 0 | -64 | 38 | 5008 | 6.12 | <.001 |
|  | Lateral occipital cortex | 46 | -64 | 46 | 990 | 5.75 | < .001 |
|  | Frontal pole / Middle frontal gyrus | -40 | 40 | 36 | 623 | 4.76 | < .001 |
|  | Lateral occipital cortex | -50 | -64 | 42 | 501 | 4.36 | < .001 |
|  | Frontal pole | 40 | 54 | -8 | 397 | 4.75 | < .001 |
|  | Middle frontal gyrus | 46 | 28 | 34 | 351 | 4.31 | < .001 |
|  | Anterior cingulate / subcallosal cortex | 0 | 32 | -2 | 287 | 4.29 | < .001 |
|  | Middle/inferior temporal gyrus | 62 | -12 | -30 | 261 | 3.94 | < .001 |
|  | Postcentral gyrus | -44 | -20 | 40 | 176 | 4.44 | < .001 |
|  | Middle temporal gyrus | 66 | -26 | -16 | 150 | 4.14 | < .001 |
|  | Right hippocampus | 30 | -28 | -10 | 134 | 4.86 | .002 |
|  | Pre-/post-central gyrus | -12 | -32 | 64 | 100 | 4.41 | .009 |
|  | Central opercular cortex | -58 | -20 | 18 | 90 | 3.87 | .016 |
|  | Parietal operculum cortex | -48 | -36 | 26 | 80 | 4.29 | .029 |
| *Association with dimensional measures* |  |  |  |  |  |  |  |
| Threat adversity | No suprathreshold clusters |  |  |  |  |  |  |
| Deprivation adversity | No suprathreshold clusters |  |  |  |  |  |  |
| General distress | No suprathreshold clusters |  |  |  |  |  |  |
| Fears | No suprathreshold clusters |  |  |  |  |  |  |
| Anhedonia-apprehension | Supramarginal / postcentral gyrus | 40 | -28 | 36 | 80 | 4.21 | .029 |

***Table S4.*** Multi-level model estimates for each face type, separated by threat and reward circuitry ROIs and by adversity dimension (threat or deprivation), * *p* < .05. Effects presented here are *including* outliers

|  | **Fear > Scrambled** | | **Sad > Scrambled** | | **Happy > Scrambled** | |
| --- | --- | --- | --- | --- | --- | --- |
|  | Estimate | *p* | Estimate | *p* | Estimate | *p* |
| *Threat circuitry ROIs* | |  |  |  |  |  |
| (Intercept) | .16 | .247 | .34 | .010* | .20 | .173 |
| Adversity: Threat | .02 | .022* | .01 | .102 | .01 | .532 |
| General Distress | -.09 | .214 | -.04 | .559 | -.00 | .984 |
| Anhedonia-apprehension | .03 | .693 | .03 | .639 | .04 | .616 |
| Fears | -.12 | .098 | -.07 | .287 | -.05 | .494 |
| Gender | -.17 | .215 | -.15 | .236 | -.12 | .424 |
| *Reward circuitry ROIs* | |  |  |  |  |  |
| (Intercept) | .19 | .159 | .04 | .769 | .18 | .220 |
| Adversity: Deprivation | .03 | .017* | .03 | .016* | .03 | .026* |
| General Distress | .00 | .996 | -.04 | .609 | -.04 | .602 |
| Anhedonia-apprehension | -.04 | .622 | -.08 | .356 | -.13 | .112 |
| Fears | -.01 | .894 | .03 | .735 | -.03 | .657 |
| Gender | -.31 | .014 | -.16 | .270 | -.16 | .249 |

Supplementary references

Andersson, J., Jesper, L., Jenkinson, M., & Smith, S. (2007) Non-linear optimisation. *FMRIB Technical Support TR07JA1*,.

Fullana, M.A., Harrison, B.J., Soriano-Mas, C., Vervliet, B., Cardoner, N., Àvila-Parcet, A., & Radua, J. (2016) Neural signatures of human fear conditioning: an updated and extended meta-analysis of fMRI studies. *Mol Psychiatry*, **21**, 500–508.

Jenkinson, M. (2002) Improved Optimization for the Robust and Accurate Linear Registration and Motion Correction of Brain Images. *NeuroImage*, **17**, 825–841.

Jenkinson, M. & Smith, S. (2001) A global optimisation method for robust affine registration of brain images. *Medical Image Analysis*, **5**, 143–156.

Lutkenhoff, E.S., Rosenberg, M., Chiang, J., Zhang, K., Pickard, J.D., Owen, A.M., & Monti, M.M. (2014) Optimized Brain Extraction for Pathological Brains (optiBET). *PLoS ONE*, **9**, e115551.

Oldham, S., Murawski, C., Fornito, A., Youssef, G., Yücel, M., & Lorenzetti, V. (2018) The anticipation and outcome phases of reward and loss processing: A neuroimaging meta-analysis of the monetary incentive delay task. *Human Brain Mapping*, **39**, 3398–3418.

Smith, S.M. (2002) Fast robust automated brain extraction. *Human Brain Mapping*, **17**, 143–155.

Tustison, N.J., Avants, B.B., Cook, P.A., Yuanjie Zheng, Egan, A., Yushkevich, P.A., & Gee, J.C. (2010) N4ITK: Improved N3 Bias Correction. *IEEE Transactions on Medical Imaging*, **29**, 1310–1320.

Tziortzi, A.C., Searle, G.E., Tzimopoulou, S., Salinas, C., Beaver, J.D., Jenkinson, M., Laruelle, M., Rabiner, E.A., & Gunn, R.N. (2011) Imaging dopamine receptors in humans with [11C]-(+)-PHNO: Dissection of D3 signal and anatomy. *NeuroImage*, **54**, 264–277.

Zhang, Y., Brady, M., & Smith, S. (2001) Segmentation of brain MR images through a hidden Markov random field model and the expectation-maximization algorithm. *IEEE Transactions on Medical Imaging*, **20**, 45–57.
